# Reduced reward anticipation in youth at high-risk for unipolar depression: A preliminary study

**DOI:** 10.1016/j.dcn.2013.11.005

**Published:** 2013-12-12

**Authors:** Thomas M. Olino, Dana L. McMakin, Judith K. Morgan, Jennifer S. Silk, Boris Birmaher, David A. Axelson, Douglas E. Williamson, Ronald E. Dahl, Neal D. Ryan, Erika E. Forbes

**Affiliations:** a University of Pittsburgh, United States; b University of Texas Health Science Center, United States; c University of California-Berkeley, United States

**Keywords:** Depression, High-risk, Reward function, Positive affect, fMRI

## Abstract

Offspring of depressed parents are at risk for depression and recent evidence suggests that reduced positive affect (PA) may be a marker of risk. We investigated whether self-reports of PA and fMRI-measured striatal response to reward, a neural correlate of PA, are reduced in adolescent youth at high familial risk for depression (HR) relative to youth at low familial risk for depression (LR). Functional magnetic resonance imaging assessments were conducted with 14 HR and 12 LR youth. All youth completed an ecological momentary assessment protocol to measure PA in natural settings and a self-report measure of depression symptomatology. Analyses found that HR youth demonstrated lower striatal response than LR youth during both reward anticipation and outcome. However, after controlling for youth self-reports of depression, HR youth demonstrated lower striatal response than LR youth only during reward anticipation. No significant differences were found between HR and LR youth on subjective ratings of PA or depressive symptoms. Results are consistent with previous findings that reduced reward response is a marker of risk for depression, particularly during reward anticipation, even in the absence of (or accounting for) disrupted subjective mood. Further examinations of prospective associations between reward response and depression onset are needed.

## Highlights

• Depression is characterized by reduced neural response to reward, particularly in the ventral striatum.
• Few studies have examined if alterations in reward functioning are present before the onset of depression.
• Youth at high- and low-familial risk for depression completed a reward task during a fMRI scan.
• High-risk youth had significantly less ventral striatal reactivity than low-risk youth during reward anticipation.
• Reward functioning is altered in individuals at high-familial risk for depression before the onset of the disorder.

## 1 Introduction

Offspring of depressed parents are at risk for developing depressive disorders (Lieb et al., 2002, Hammen et al., 2004, Klein et al., 2005, Goodman et al., 2011) and other functional impairments (Beardslee et al., 1998, Lewinsohn et al., 2005). Rich theoretical perspectives outline potential mechanisms of risk, including biological and psychosocial factors (Goodman and Gotlib, 1999, Goodman and Gotlib, 2002). However, there remains only a modest literature examining specific neural processes through which parental depression is related to offspring depression. One potential mechanism of transmission or vulnerability marker of risk in youth is positive affect (PA) that includes subjective experience and neural functioning.

Positive affect plays a central role in depression as diminished experience of interest and/or pleasure is a cardinal symptom of the disorder and has been linked to risk for onset, recurrence, and likelihood of remission (Pine et al., 1999, Kasch et al., 2002, McMakin et al., 2012). Studies of PA in the context of depression have relied on indices of personality/temperament (including extraversion and positive emotionality; Compas et al., 2004, Clark, 2005, Kotov et al., 2010), affective experience (including positive affect; Laurent et al., 1999, Joiner and Lonigan, 2000, McMakin et al., 2009), and neural functioning (including response to reward; for a meta-analytic review see Zhang et al., 2013). These affective responses are active when working toward or achieving goals and experiencing PA states. However, most of these investigations have relied on cross-sectional comparisons between depressed and non-depressed participants. Thus, these studies cannot speak to whether altered PA is a predictor of onset, a correlate of the disorder episode, or a consequence (i.e., scar) of experiencing a depressive episode. To determine whether low PA is associated with developing depression, prospective studies are needed. However, it is also important to examine whether low PA is associated with established risk factors for depression, notably family history of depression.

Supportive evidence for reduced PA in youth at familial risk for depression comes from lines of work involving behavioral displays of PA. Offspring of depressed parents demonstrate lower levels of PA than offspring of parents without a history of depression. For example, Durbin et al. (2005) found that three-year old children of mothers with a history of depression demonstrated lower levels of PA, indexed by smiling, laughter, and interest in exploration of stimuli, across a series of structured laboratory tasks. In addition, Olino et al. (2011) examined longitudinal changes in laboratory assessed PA in youth, primarily indexed by smiling and laughter, of depressed and non-depressed mothers spanning late infancy through age 9. The authors found that offspring of depressed mothers demonstrated significantly lower levels of PA across childhood than offspring of mothers without a history of depression. Thus, these studies highlight that behavioral displays of positive affect differentiate between young children at high- and low-risk for depression. However, fewer studies have examined similar questions beyond childhood; thus, it is unclear if similar associations continue to be present in adolescence. Rather than relying on behavioral observations, adolescents can complete reports of affect in their naturally occurring environments that can improve ecological validity of measurement. Further, these results are suggestive that neural mechanisms of PA would also be affected.

At a biological level of analysis, PA is often described as influencing or being influenced by striatal function (among other functions, including avoidance of punishment; Forbes, 2009, Haber and Knutson, 2010). In particular, the ventral striatum (VS), inclusive of the nucleus accumbens, is responsive when pursuing, encountering, and seeing cues of multiple classes of reinforcers, including drugs, food, and money (Berridge and Robinson, 2003, Nestler and Carlezon, 2006, Haber and Knutson, 2010). Indeed, experimental manipulations of the VS, by administering amphetamines, have produced positive affective states in healthy participants (Drevets et al., 2001). In addition, adolescent reports of PA in naturalistic environments have previously been reported to be associated with ventral striatal response during reward anticipation and receipt (Forbes et al., 2009, Forbes et al., 2010). Results of studies of reward response across adolescence has provided mixed findings, with some studies finding reduced (e.g., Forbes et al., 2010) and others finding increased (e.g., Galvan et al., 2006) striatal response across development. Despite these differences, however, most researchers interpret the results as indicating that adolescence is a period marked by greater reward responsiveness than during childhood or adulthood.

While much of the work delineating striatal response to reward has focused on healthy populations, a number of recent studies examined the influence of depression on reward function. These studies have typically found that individuals with depression have lower levels of striatal response than individuals without depression (Nestler and Carlezon, 2006, Forbes et al., 2009, Pizzagalli et al., 2009, Smoski et al., 2011, Dichter et al., 2012) and are summarized by a recent meta-analysis (Zhang et al., 2013). However, given the possibility of state influences or scarring effects of depression on striatal function, these studies cannot speak to whether altered reward functioning is a cause, correlate, or consequence of depression. Studies focusing on individuals at-risk for depression are necessary to identify if altered reward-related brain functioning is present in individuals before depressive disorder onsets.

A small number of studies have examined neurobiological response to positively valenced stimuli and rewards in youth at high risk for depression. Monk et al. (2008) found that youth at high-risk for depression demonstrated lower levels of nucleus accumbens response while viewing happy facial expressions than low-risk youth. However, as the task involved passive viewing of faces, it is unclear whether striatal response reflected a motivational tendency toward reward or general response to positive valence. Gotlib et al. (2010) examined response to a monetary incentive task in girls at-risk for depression. The authors reported that high-risk girls demonstrated lower putamen response than low-risk girls during anticipation of reward, but did not find differences during the receipt of rewards. The results reported by Gotlib et al. are suggestive that differences may vary between anticipation and consummatory phases of rewards (Davidson, 1998, Berridge and Robinson, 2003). However, as the high-risk girls in Gotlib et al. had significantly higher (albeit sub-syndromal) levels of depressive symptoms than the low-risk girls, it is possible that current symptoms, rather than high-risk status, may have driven the results.

This seminal work examining differences between youth at high- and low-familial risk for depression has provided support for the hypothesis that reward-system alterations are present before the onset of depression. Indeed, some have hypothesized that low PA, either conceptualized as hypohedonia (Meehl, 1975, Meehl, 2001) or attenuated reward function (Hasler et al., 2004), are endophenotypes for depression. That is, attenuated PA responses would be present before, during, and following episodes, and are familial, among other considerations for characteristics being endophenotypes (Gottesman and Gould, 2003). However, there are still many questions to be addressed. One particular need of this literature is to examine multiple aspects of PA in the same sample. No previous study included both subjective reports of positive affect, particularly in ecologically valid contexts, and neural probes of reward functioning. Further, as youth at high-risk for depression often demonstrate higher levels of depressive symptoms than peers, it is important to consider state effects of youth symptoms on brain functioning. This can provide additional leverage for understanding whether family history is directly or indirectly influencing youth outcomes.

The present study examines differences in PA and reward-related brain functioning in youth at high- and low-risk for depression. We further examine if observed group differences are accounted for by youth reports of depressive symptoms. We hypothesize that offspring at high-risk for depression will demonstrate reduced PA and reward-related brain functioning relative to low-risk offspring and that these differences will persist after accounting for individual differences in subjective reports of depressive symptomatology.

## 2 Material and methods

### 2.1 Participants

Participants come from a larger study of pediatric affective disorders (*n* = 78; age 8–17). The present report focused on the first functional magnetic resonance imaging (fMRI) assessment of healthy youth who were reported on in a previously published report (Forbes et al., 2009). All youth were considered psychiatrically healthy, but varied on family history of depression. There were 26 youth included, with a mean age of 15.72 (SD = 2.82); 73.1% (*n* = 19) were female; and 92.3% (*n* = 24) were Caucasian. Socioeconomic status was assessed using the Hollingshead Index (Hollingshead, 1975).

Family history of psychopathology was assessed by masters-level clinicians with the Structured Clinical Interview for DSM-IV (First et al., 1996) for parents who were assessed in person (nearly always the youth's mother). For family members not directly assessed, informant reports on first- and second-degree relatives were collected using the Family History-Research Diagnostic Criteria (FH-RDC; Endicott et al., 1978). Informant reports were almost always provided by the youth participant's mother. Final diagnoses were determined via consensus checks with senior psychiatrists on the research team. Based on this information, youth were classified as having a family history of mood disorders (*n* = 14) and no family history of unipolar depressive disorders (*n* = 12). High-risk youth had at least two first-degree relatives or one first-degree and two second-degree relatives with a history of unipolar depression (i.e., HR youth). Thus, these youth were at very high familial-risk for depression. Low-risk youth themselves had no history of case-level psychopathology and no psychopathology in first- or second-degree family members (i.e., LR youth). Youth in HR and LR groups did not differ on gender, age, or SES (see Table 1).

**Table 1:** Demographic characteristics of high- and low-risk youth.

	Low-risk	High-risk	*t*/*χ*^2^
Female^a^	8 (66.7%)	11 (78.6%)	.46
Age^b^	15.58 (2.59)	15.85 (3.09)	−0.23
SES^b^, ^c^	46.20 (9.42)	39.94 (9.42)	.85
Youth depressive symptoms^b^	1.92 (2.57)	3.14 (2.90)	1.12
PA^b^	3.28 (.54)	3.29 (.63)	.03

a Indicates that *n* (and percentage) for each group is presented and a *χ*^2^ statistic is computed for the test of statistical significance.

b Indicates that the mean (and standard deviation) for each group is presented and a *t*-statistic is computed for the test of statistical significance.

c Due to violation of the equal variance assumption, the *t*-statistic was computed based on unequal variances. Youth depressive symptoms were assessed using the Mood and Feelings Questionnaire. High-risk status was defined as having a family history of unipolar depression in at least two first-degree relatives or one first-degree and two second-degree relatives (vs. low-risk status; defined as having no family history of depression in either first- or second-degree relatives). PA is positive affect measured using ecological momentary assessment procedures.

### 2.2 Measures

Youth completed the Mood and Feelings Questionnaire (MFQ; Angold et al., 1995) to assess current levels of depressive symptomatology. Overall, the sample had low levels of symptoms (*M* = 2.58, SD = 2.77; range 0–9 [for both HR and LR youth]). Thus, on average, youth were not endorsing clinically significant problems (Burleson Daviss et al., 2006).

Youth completed an ecological momentary assessment (EMA) protocol to assess PA. Self-reports of PA were collected via cell phone in natural settings (see Silk et al., 2011 for more details). Youth were contacted on 12 occasions over the course of 4 days (Friday through Monday). Calls did not take place during school hours on school days. The protocol was repeated at baseline and 1, 3, 5, and 7 weeks after baseline. Data were missing or incomplete for 5% of calls. Calls were administered by research associates, who also ensured that participants understood the rating scales and vocabulary of the items. For each call, youth provided responses to items from the Positive Affect-Negative Affect-Child version (PANAS-C; Laurent et al., 1999), which has strong psychometric properties. The full instrument was administered once per day and a subset of positive affect items (happy, joyful, energetic, excited) was administered at all other calls. All items were rated on a five-point Likert scale ranging from ‘very slightly or not at all’ to ‘extremely’. No significant changes were found in reports of PA across weeks. Thus, a single PA composite was computed by averaging PA items across all available assessments. This was done to derive the most comprehensive index.

Youth also completed an fMRI assessment session that included a card guessing paradigm previously used with youth (Forbes et al., 2009, Forbes et al., 2010) and adults (Lahey et al., 2012). This fMRI paradigm consistently probes striatal response to feedback associated with monetary reward. Each trial includes both an anticipation and outcome period, and participants received win, loss, or no-change feedback for each trial. The participants were told that their performance would determine a monetary reward to be received after the scan.

Trials were presented in pseudorandom order with predetermined outcomes. During each 27-s trial, the participants had 3 s to guess, through button press, whether the value of a visually presented card with a possible value of 1–9 was higher or lower than 5 (index and middle finger of a scanner-compatible glove, respectively). After a choice was made, the trial type (reward or loss) was presented visually for 12 s (anticipation). This was followed by the “actual” numerical value of the card (500 ms); outcome feedback (a green upward-facing arrow for win, a red downward facing arrow for loss, or a yellow circle for neutral feedback; 500 ms); and a crosshair presented for 11 s (outcome). The last 3 s of the outcome phase was treated as a baseline, inter-trial interval. Thus, for the analyses, all outcome phases were treated as 8 s intervals. Trials were presented in 4 runs, with 12 trials per run, and a balanced number of trial types within runs.

The participants were told that they would receive $1 for each win, lose 50 cents for each loss, and experience no earnings change for neutral outcomes. The participants were unaware of the fixed outcome probabilities and were led to believe that performance would determine net monetary gain. The participants’ engagement and motivation to perform well were maintained by verbal encouragement during practice and between runs. In order to maximize the information about striatal response and sample size, striatal response during reward anticipation and outcome was averaged across all available runs. Thus, the number of available runs varied across participants. Across all participants, 16 (61.5%) had all four runs, 2 (7.7%) had three runs, 5 (19.2%) had two runs, and 3 (11.5%) had only one run. However, the mean number of available runs did not differ between HR and LR youth (*t* = .58, *p* = .58).

### 2.3 BOLD fMRI acquisition, processing, and analysis

Each participant was scanned using a Siemens 3T Allegra scanner. BOLD functional images were acquired with a gradient echo planar imaging sequence and covered 34 axial slices (3 mm thick) beginning at the cerebral vertex and encompassing the entire cerebrum and the majority of the cerebellum (TR/TE = 2000/25 ms, field of view = 20 cm, matrix = 64 × 64). Scanning parameters were selected to optimize BOLD signal quality while maintaining a sufficient number of slices to acquire whole-brain data. Before the collection of fMRI data for each participant, a reference echoplanar imaging scan was acquired and visually inspected for artifacts (e.g., ghosting) and good signal across the entire volume. The data from all 26 participants were clear of such problems.

Whole-brain image analysis was conducted with SPM2 (http://www.fil.ion.ucl.ac.uk/spm). For each scan, images for each participant were realigned to the first volume in the time series to correct for head motion. The motion correction criterion was set at <4 mm, which is higher than that used in many fMRI studies, to maximize the size of this sample containing young people.

Realigned images were spatially normalized into Montreal Neurological Institute stereotactic space using a 12-parameter affine model, then smoothed to minimize noise and residual difference in gyral anatomy with a Gaussian filter set at 6 mm full-width at half-maximum. Voxel-wise signal intensities were ratio normalized to the whole-brain global mean.

Preprocessed data were analyzed using second-level random effects models that account for both scan-to-scan and participant-to-participant variability to determine task-specific regional responses. These group level analyses were conducted in SPM8. Analyses focused on all available data for participants. Thus, when youth had available data from multiple runs, the average activation was computed across all available runs. When only a single run was available, the single run was used in the analysis. Individual runs were not included when average movement exceeded 4 mm (or 4°) in any of six directions from the first volume.

Because a priori hypotheses concerned the role of PA in depression, analyses focused on the reward conditions. For each participant and scan, predetermined condition effects at each voxel were calculated using a *t* statistic, producing a statistical image for two contrasts: reward anticipation > baseline and reward outcome > baseline. Analyses focused on a striatal region of interest, based on the typical pattern of response in similar reward tasks, encompassing the entire bilateral ventral striatum and adjacent regions of the caudate (sphere with 20 mm radius, centered on Talairach coordinates *x* = 0, *y* = 10, *z* = −10). AlphaSim was used to estimate minimum cluster size thresholds that exceed corrected *p* < .05. For the striatal ROI, a minimum cluster size of 185 contiguous voxels was needed to exceed *p* < .05. The MFQ, EMA PA, and guessing task were all used in Forbes et al. (2009), although, here we focus solely on the healthy youth.

## 3 Results

### 3.1 Association between self-reported PA and striatal response

We examined associations between youth self-reports of PA and depression and striatal response across all participants. For anticipation, youth reports of PA were positively associated with striatal response (*k*_E_ = 261, peak voxel = 8 −6 0, *t* = 3.29, *p* < .05). Similarly, for outcome, youth reports of PA were positively associated with striatal response (*k*_E_ = 294, peak voxel = 10 2 2, *t* = 3.57, *p* < .05).

### 3.2 Differences in self-reports

Independent samples *t*-tests were conducted to examine differences in youth reports of PA and depressive symptoms between youth at high- and low-risk for depression. High-risk and low-risk youth did not significantly differ on youth reports of PA or current depressive symptoms (Table 1).

### 3.3 Differences in striatal response based on risk-status

Initial analysis examined differences in striatal activation during reward anticipation and outcome between youth at high- and low-familial risk for depression. Thus, separate *t*-tests were estimated for anticipation and outcomes. Within the specified striatal ROI, youth at high-risk for depression demonstrated significantly less activation than youth at low-risk for depression during the anticipation (*k*_E_ = 759, peak voxel Talairach = 1 9 5, *t* = 3.92, *p*_corrected_ < .05) and the outcome phase (*k*_E_ = 232, peak voxel = −8 0 6, *t* = 2.55, *p*_corrected_ < .05).

### 3.4 Association between self-reported depressive symptoms and striatal response

Next, we examined associations between self-reported depressive symptoms and striatal response and striatal response during anticipation and outcomes. Within the specified striatal ROI, depressive symptoms were significantly negatively associated with striatal response during anticipation (*k*_E_ = 411, peak voxel Talairach = −6 10 4, *t* = 3.68, *p*_corrected_ < .05) and during outcome (*k*_E_ = 507, peak voxel = 2 10 5, *t* = 5.38, *p*_corrected_ < .05).

### 3.5 Risk-status, self-reported depressive symptoms, and striatal response

**Fig. 1:**
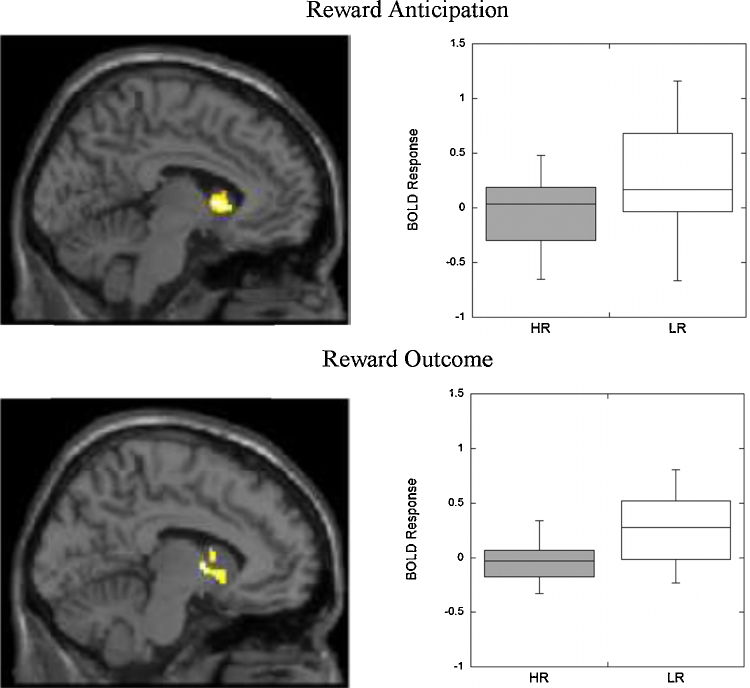
Group differences in striatal response between youth at high-risk and low-risk for depression during reward anticipation and outcome controlling for youth self-reported depressive symptoms. Top displays group differences in striatal response during reward anticipation between youth at high-risk and low-risk for depression. Bottom displays group differences in striatal response during reward outcome between youth at high-risk and low-risk for depression. Based on AlphaSim corrections, this difference did not survive multiple comparison corrections. Boxplots provide descriptive information about group differences. Images are centered on coordinates presented in Table 2. Statistical tests are displayed in Table 2.

**Fig. 2:**
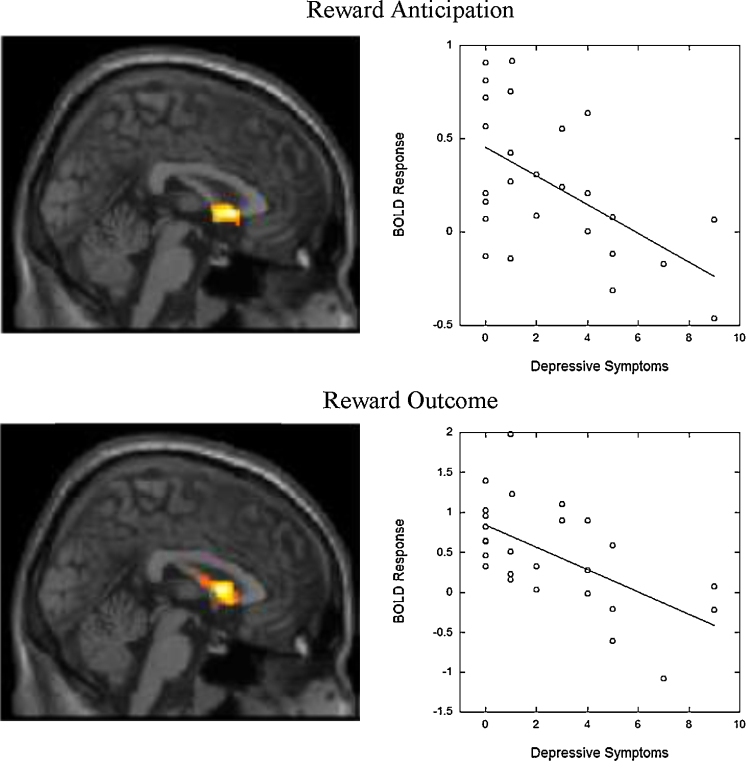
Associations between self-reported depressive symptoms and striatal response during anticipation and outcome controlling for risk status (high- vs. low-risk). Top displays negative associations between youth self-reported depressive symptoms and striatal response during reward anticipation when controlling for risk status. Bottom displays negative associations between youth self-reported depressive symptoms and striatal response during reward outcome when controlling for risk status. Boxplots provide descriptive information about group differences. Images are centered on coordinates presented in Table 2. Statistical tests are displayed in Table 2.

Finally, we estimated models that included associations between risk status and youth reports of depressive symptoms in a multiple regression model in SPM predicting striatal response during reward anticipation and outcome. For reward anticipation, high-risk (vs. low-risk) status (Fig. 1, top panel) and youth reports of higher levels of symptoms (Fig. 2, top panel) were both associated with lower levels of striatal response (Table 2). For reward outcome, youth reports of higher levels of symptoms were each associated with lower levels of striatal response (Fig. 2, bottom panel). However, high-risk (vs. low-risk) status was no longer significantly associated with lower levels of striatal response (Fig. 1, bottom panel; alpha simulation cluster size, *p* = .15). Parallel results were found with whole-brain analytic methods (see Supplementary Table 1). In these whole-brain analyses (*p*_uncorrected_ < .001, *k*_E_ ≥ 25), youth symptoms were associated with reduced response in the dorsal medial PFC and HR status was associated with reduced response in the VS, caudate, and precuneus. No clusters survived the threshold for either youth symptoms or risk-status for the outcome phase. Finally, we examined the same sets of analyses for participants who had complete data. The interpretations were substantively identical.

**Table 2:** Results of multivariate analysis of familial risk status and youth depressive symptoms.

	Cluster size	Coordinates			Statistic
	*k*_E_	*x*	*y*	*z*	*t*
**Anticipation**					
Youth depressive symptoms	245	−7	7	8	3.04
High-risk	645	0	9	5	3.83
**Outcome**					
Youth depressive symptoms	388	0	7	10	4.11
High-risk	126	−8	0	6	2.47

Analyses focused on a striatal region of interest defined as a sphere with 20 mm radius, centered on Talairach coordinates *x* = 0, *y* = 10, *z* = −10. Cluster sizes exceeding 185 are significant at *p* < .05 based on AlphaSim corrections. The identified clusters indicate where youth depressive symptoms were *negatively* correlated with striatal response and where high-risk youth demonstrate lower striatal response than low-risk youth. Youth depressive symptoms were assessed using the Mood and Feelings Questionnaire. High-risk status was defined as having a family history of unipolar depression in at least two first-degree relatives or one first-degree and two second-degree relatives (vs. low-risk status; defined as having no family history of depression in either first- or second-degree relatives).

## 4 Discussion

Arguments have been made that low levels of PA may serve as a vulnerability marker or endophenotype for depression (Meehl, 1975, Meehl, 2001, Hasler et al., 2004). Previous work using behavioral observations of young children have suggested that low levels of PA differentiates between youth at high- and low risk for unipolar depression (Durbin et al., 2005, Olino et al., 2011). There have been some attempts to identify parallel differences at the neural level using fMRI. In older children and adolescents, youth at high-risk for depression demonstrate lower levels of striatal response when winning money (Gotlib et al., 2010) and viewing happy faces (Monk et al., 2008) than youth at low-risk for depression. An important consideration in interpreting the work of Gotlib et al. is that, although no youth participants had a history of clinical depression, girls at high-risk for depression demonstrated significantly higher levels of symptoms than the girls at low-risk. The present study extends this work by examining both subjective reports of PA and brain-based reward responses during a monetary incentive task in the same sample. In addition, we also examine the influence of familial risk status on striatal response after accounting for youth reports of sub-syndromal depressive symptoms. Finally, as our high- and low-risk youth did not differ on their reports of depressive symptoms, our results highlight the role of family history on PA and striatal response.

We hypothesized that high-risk youth would demonstrate attenuated reward-related brain functioning, as indexed by striatal response, than low-risk youth. In initial analyses focusing solely on familial risk status, we found that, indeed, high-risk youth did demonstrate lower levels of striatal response than low-risk youth during reward anticipation and outcome. In follow-up analyses, we examined whether the influence of familial risk status remained associated with striatal response beyond the influence of youth self-reported depressive symptoms. In these analyses, familial high-risk status continued to be associated with lower levels of striatal response during reward anticipation. However, after controlling for youth PA and depressive symptoms, risk status was no longer significantly associated with striatal response during reward outcome. Although the present study design cannot distinguish between biological and environmental mechanisms of relating family history of depression to youth brain function, these results suggest that family history of depression conveys unique influence on striatal response that is not accounted for by current state of affective disturbance.

The presence of attenuated striatal response among these youth is quite impressive. Our participants’ age was approximately 16 years, and high-risk youth often have earlier ages of depression onset than low-risk youth. Thus, our high-risk youth might represent a resilient group and these youth are demonstrating a vulnerability marker without clinically significant depressive symptoms.

Our results found a discrepancy in differences across levels of risk during reward anticipation and outcome is interesting and consistent with the findings reported by Gotlib et al. (2010). Various models have contrasted phases of reward (Davidson, 1998, Berridge and Robinson, 2003) and some have argued that the core deficit in depression is an attenuated approach motivation tendency (Davidson, 1998). Indeed, some work relying on self-report measures find that anticipatory, but not consummatory, PA is associated with symptoms of depression (Gard et al., 2006). The current work supports this conjecture. However, these results are inconsistent with the recent meta-analytic work finding reduced striatal response during both anticipation and outcome phases of reward (Zhang et al., 2013). This may suggest a developmental progression of attenuated anticipation of rewards before the onset of depressive disorders that is followed by dampened responses to receipt of rewards during episodes. That is, as individuals seek rewards less consistently and also experience low, stable, impairing levels of PA, their response to those rewards becomes weaker over time.

Previous work (Gotlib et al., 2010) reported differences in reward anticipation and outcome in an all-female sample of adolescents in dorsal striatal (i.e., putamen) and anterior cingulate regions in the Monetary Incentive Delay task (MID; Knutson et al., 2008). It is possible that task related differences may have influenced the pattern of results, with greater motor and attention demands in the MID. In addition, the MID includes a true performance component (i.e., reaction time) that determines success, as opposed to relying on predetermined outcomes.

In contrast to the striatal response findings, youth did not significantly differ on PA when assessed in natural environments. The lack of convergence across methods was not due to the measures assessing different constructs as PA was associated with striatal response. Thus, we speculate on this discrepancy. It is likely that youth seek out environments that they enjoy, particularly during adolescence, and, in these environments, youth at high- and low-risk for depression do not differ on PA. Alternatively, EMA focused specifically on current or very recent past affective experiences and was intended to narrow recall biases. Thus, moment-to-moment affect ratings may be less suspect to biases in characterizing PA as reduced relative to lab-based measures of affect, personality, or temperament (Kotov et al., 2010). Finally, our EMA PA measure may have largely reflected consummatory (i.e., outcome-based) PA. Thus, our results could be consistent across methods.

These results also have important implications for understanding the magnitude of differences between depressed and healthy youth. Previously, Forbes et al. (2009) reported on differences between depressed youth and the healthy youth described here, inclusive of both the high- and low-risk youth. However, we find that high-risk youth demonstrate lower levels of striatal response than low-risk youth. Thus, by including high-risk youth with less striatal response in the healthy group, the previously reported findings appear to underestimate the magnitude of the differences between depressed and healthy youth.

The present study relied on a cross-sectional design to identify whether youth at high-risk for depression demonstrated significantly less striatal response during phases of reward relative to low-risk youth. The findings are highly suggestive that alterations in reward processing are present in high-risk youth before the onset of disorder. Thus, this may reflect a promising marker of depression risk (i.e., an endophenotype; Hasler et al., 2004). However, much longitudinal work is necessary to understand the broader context of this work. First, there is little available data concerning prospective associations between reward-related brain functioning and the onset of unipolar depressive disorder. Some promising work finds that reward-related brain functioning is prospectively associated depression. Bress et al. (2013) found that the feedback negativity component assessed during a monetary incentive task was predictive of depression onset in adolescent girls. Similarly, but using neuroimaging, Morgan et al. (2013) found that, among older adolescents, reduced striatal response during a monetary reward task was associated with increases in depressive symptoms over the course of two years. Second, if reward-related brain functioning is associated with depression onset, it will be important to consider the longitudinal trends in underlying neurobiological changes in reward processing. While there are some available data concerning differences across developmental status among healthy participants (Ernst et al., 2005, Galvan et al., 2006, Forbes et al., 2010), there is an absence of data on how developmental status influences or is influenced by risk-status. Thus, investigations of longitudinal changes in brain-functioning (and behavioral indicators of approach motivation) in high-risk youth may be important for identifying individuals at very high-risk or those likely to be resilient against adverse outcomes. Third, important questions remain about biological (e.g., genetics, temperament) and environmental influences (e.g., parenting) that result in observed cross-sectional differences. Thus, important longitudinal work with an emphasis on young children will be crucial for elucidating these processes. Fourth, additional questions remain concerning responses to various types of rewards. Our work and that of Gotlib et al. (2010) relied on monetary incentives, whereas Monk et al. (2008) studied relied on facial stimuli. More recently, investigators have pursued social rewards in the form of positive and negative feedback (Guyer et al., 2008, Davey et al., 2009, Silk et al., 2012). It is crucial that this work is investigated in the context of depression, particularly in adolescence when changes in social contexts and peer relationships are substantial.

The present study examined a well-characterized sample of youth at high- and low-familial risk for depression using a task that reliably activates reward circuitry. In addition, we controlled for youth self-reports of depressive symptoms as a means of identifying unique influences of risk status and personal depression severity on reward anticipation. However, the study had some limitations. First, the sample was small, and, thus, replication of this work with larger samples is needed. This is particularly important as the attenuated influence of risk-status on striatal response may have been due to power loss when adding covariates to a small sample. Second, high-risk youth all had a strong family history of unipolar depression, suggesting genetic and biological transmission of altered reward functioning. However, we could not test this explicitly. In addition, the informant for family history of psychopathology was largely from a single source. Thus, future work would benefit from assessing psychopathology directly with additional family members. Third, although groups did not differ on a number of characteristics, some differences had moderately sized effects (e.g., depressive symptoms). Thus, future work should incorporate these characteristics into their investigations. Fourth, due to excessive movement on specific task runs, participants varied in the number of runs that contributed to the analyses. Thus, the precision of measured brain response during anticipation and outcome phases of the task would vary across participants. However, the number of available runs was similar for high- and low-risk youth and results were substantively the same when analyses included only participants who had complete data. Finally, the outcome phase and baseline inter-trial intervals were fully adjacent and may not have been sufficiently distinct to dissociate response to each type of event. Future methodological work is needed to examine how to better discriminate between these phases for this task (e.g., decreasing the length of the outcome phase and introducing a jittered inter-trial interval).

In sum, the present study found that youth at high-risk for depression demonstrated lower levels of reward response during reward anticipation relative to youth at low-risk for depression. This was found after accounting for current PA and depressive symptomatology. However, further longitudinal work is necessary to evaluate the developmental and clinical implications of these cross-sectional differences.

## Conflict of interest

Boris Birmaher has or will receive royalties for publications from Random House Inc. (*New hope for children and teens with bipolar disorder*) and Lippincott Williams & Wilkins (*Treating Child and Adolescent Depression*). All other authors report no conflicts.
