# Supplementary material for: Reduced reward anticipation in youth at high-risk for unipolar depression: A preliminary study
Source: Dev Cogn Neurosci. 2013 Dec 12;8:55–64. doi: 10.1016/j.dcn.2013.11.005 (PMC3960320; doi:10.1016/j.dcn.2013.11.005)
Supplement: Supplementary file 1 [file mmc1.docx]

Supplemental material.

We also examined whole-brain analyses for our final models that included both risk status and self-reported depressive symptoms predicting response. These analyses were thresholded at an uncorrected *p* < .001 and a minimum cluster size of 25 contiguous voxels. Analyses were conducted for anticipation and outcome phases of the task.

Supplemental Table 1. Results of whole-brain multivariate analysis of familial risk status and youth depressive symptoms during anticipation and outcome phases.

|  | Cluster Size | Coordinates | | | Statistic |
| --- | --- | --- | --- | --- | --- |
| Anatomical Region | k_E_ | x | y | z | *t* |
|  | **Anticipation** | | | | |
| **Youth Depressive Symptoms** |  |  |  |  |  |
| dmPFC | 101 | 2 | 33 | 48 | 6.53 |
| **High-Risk (vs. Low-Risk) Status** | |  |  |  |  |
| Caudate | 28 | -18 | 21 | -1 | 5.22 |
| Caudate | 27 | 8 | 20 | 12 | 4.80 |
| Ventral Striatum | 46 | 2 | 12 | -1 | 4.59 |
| Precuneus | 26 | -26 | -51 | 58 | 4.14 |
|  | **Outcome** | | | | |
| **Youth Depressive Symptoms** |  |  |  |  |  |
| None |  |  |  |  |  |
| **High-Risk (vs. Low-Risk) Status** | |  |  |  |  |
| None |  |  |  |  |  |

The identified clusters indicate where youth depressive symptoms were *negatively* correlated with striatal response and where high-risk youth demonstrate lower striatal response than low-risk youth. Youth Depressive Symptoms were assessed using the Mood and Feelings Questionnaire. High-risk status was defined as having a family history of unipolar depression had at least two first-degree relatives or one first-degree and two second-degree relatives with a history of unipolar depression (vs. low-risk status; defined as having no family history of depression in either first- or second-degree relatives).
